# Enhanced pain-related conditioning for face compared to hand pain

**DOI:** 10.1371/journal.pone.0234160

**Published:** 2020-06-19

**Authors:** Katharina Schmidt, Katarina Forkmann, Sigrid Elsenbruch, Ulrike Bingel

**Affiliations:** 1 Department of Neurology, University Hospital Essen, University Duisburg-Essen, Essen, Germany; 2 Institute of Medical Psychology and Behavioral Immunobiology, University Hospital Essen, University Duisburg-Essen, Essen, Germany; 3 Department of Medical Psychology and Medical Sociology, Ruhr University Bochum, Bochum, Germany; Swansea University, UNITED KINGDOM

## Abstract

Pain is evolutionarily hardwired to signal potential danger and threat. It has been proposed that altered pain-related associative learning processes, i.e., emotional or fear conditioning, might contribute to the development and maintenance of chronic pain. Pain in or near the face plays a special role in pain perception and processing, especially with regard to increased pain-related fear and unpleasantness. However, differences in pain-related learning mechanisms between the face and other body parts have not yet been investigated. Here, we examined body-site specific differences in associative emotional conditioning using electrical stimuli applied to the face and the hand. Acquisition, extinction, and reinstatement of cue-pain associations were assessed in a 2-day emotional conditioning paradigm using a within-subject design. Data of 34 healthy subjects revealed higher fear of face pain as compared to hand pain. During acquisition, face pain (as compared to hand pain) led to a steeper increase in pain-related negative emotions in response to conditioned stimuli (CS) as assessed using valence ratings. While no significant differences between both conditions were observed during the extinction phase, a reinstatement effect for face but not for hand pain was revealed on the descriptive level and contingency awareness was higher for face pain compared to hand pain. Our results indicate a stronger propensity to acquire cue-pain-associations for face compared to hand pain, which might also be reinstated more easily. These differences in learning and resultant pain-related emotions might play an important role in the chronification and high prevalence of chronic facial pain and stress the evolutionary significance of pain in the head and face.

## Introduction

Pain is evolutionarily hardwired to signal potential danger and threat. Thus, learning about signals or situations that predict pain is a highly adaptive response as it allows to prepare for upcoming pain and to effectively cope with it, for instance by initiating protective behavior [1–3]. Pain signals might also initiate maladaptive responses such as increased negative emotions, e.g. fear of pain [4] as described in the fear-avoidance model of chronic pain [2, 5]. These anticipatory negative emotions might lead to an aggravation of pain experiences [6, 7]. Recent research suggests that the development and maintenance of chronic pain might be associated with alterations in pain-related learning of negative emotions [1, 8]. Alterations in different learning processes (e.g. enhanced acquisition, reduced extinction, or enhanced reinstatement effects) might differentially contribute to the chronification of pain.

For more than 100 years classical conditioning paradigms have been used to investigate distinct learning mechanisms: *acquisition* describes the establishment of associations when an unconditioned stimulus (US), such as a painful or aversive stimulus is contingently paired with a conditioned stimulus (CS) inducing conditioned responses (CR). The acquisition of CR might depend on the characteristics of the US, such as aversiveness or salience [3]. Once US-CS associations are acquired, these might be *extinguished* when the US is no longer present. This *extinction learning* is thought to be an active, inhibitory learning process rather than just “forgetting” of CR [9], supported by phenomena like *spontaneous recovery*, i.e., a decay of the CR through the passage of time [10–12] or *reinstatement*, i.e. the retrieval of an extinguished memory after an unexpected exposure to the US [13, 14]. These learning processes have been thoroughly investigated for fear-learning in anxiety disorders [15], but only recently has research started to study pain-related learning mechanisms in experimental and chronic pain (e.g., [16–18]) suggesting enhanced conditioned pain-related fear, impaired differential learning, and increased fear generalization of CS in chronic pain. There are only few studies examining pain-related spontaneous recovery [19] or reinstatement of previously extinguished CR reporting inconclusive findings. While some report increased behavioral and neural responses during the reinstatement of CR [20–22], others did not observe such reinstatement effects [23].

Differences in pain-related learning depending on the site of stimulation have only sparsely been investigated, despite its potential clinical relevance [24]. Klinger, Matter [25] investigated differences in learning mechanisms using painful stimulation at the hand and found increased conditioned muscular responses in chronic back pain and chronic headache patients compared to healthy controls. Harvie, Meulders [24] recently compared painful US applied to different body sites. Differential fear learning in terms of startle responses, fear and expectancy ratings was observed for hand pain but not for back pain stimuli, which was related to differences in sensory acuity in these regions. Such investigations are lacking for face pain despite its high salience and clinical relevance [26].

Given the outstanding biological relevance of face pain, i.e., potential danger to vital functions, learning of cues predicting face pain might be of particular relevance and prioritized over cues signaling threat to other external body parts. Face pain is associated with increased pain-related fear compared to pain on extremities [27]. Moreover, pain applied to an extremity positioned near the head elicits strong defensive reactions [28, 29] and neural reinstatement after single trial conditioning was enhanced for face compared to hand pain [30]–results that may be explained by the inherent salience and threat of face pain stimuli. Stimulus-related differences in learning have also been suggested in other clinically relevant pain models, i.e. somatic and visceral pain [31, 32], stressing the importance of US salience for pain perception and learning of pain-related emotional responses as well as the role of the aversiveness of a US [33, 34].

In the present study, we aimed to investigate the acquisition, extinction, extinction recall, and reinstatement of emotional responses to cues predicting electrical pain stimuli applied to the face or the hand in healthy participants using a 2-day conditioning paradigm. We investigated differences in *acquisition* and *extinction* of emotional responses (i.e., change in valence) on the first day to initially neutral visual stimuli that predict face or hand pain using a within-subject design. On the second day, an *extinction recall* phase prior to the unsignaled exposure to both US (*reinstatement phase*) was included to investigate spontaneous recovery.

With regard to the different experimental phases we expected the following results for learning of US-CS associations as assessed using valence ratings:

1. Acquisition phase (day 1): enhanced emotional learning, i.e., steeper learning curve of negative valence for CS^+face^ than CS^+hand^ (non-differential).
2. Extinction phase (day 1): reduced extinction, i.e., slower decrease of negative valence ratings for CS^+face^ than CS^+hand^ (non-differential).
3. Extinction recall phase (day 2): stronger increase in negative valence for CS^+face^ than CS^+hand^ from the end of extinction to the beginning of extinction recall (i.e. increased spontaneous recovery).
4. Reinstatement test phase (day 2): stronger increase in negative valence for CS^+face^ than CS^+hand^ from the end of extinction recall to after the unsignaled exposure of both US (i.e. increased reinstatement).

## Methods

### Participants

Participants were recruited locally to participate in the study. The study had been approved by the local Ethics Committee (University of Duisburg-Essen, Germany; 16-7248-BO). All subjects gave written informed consent and received monetary compensation for study participation. Participants were free to withdraw from study participation at any time.

Based on previously reported medium to large effect sizes for interaction effects between body parts during the acquisition of conditioned fear [24], we decided to include 39 subjects participated in the study. Due to technical difficulties or no sufficiently painful sensation induced by the electrical stimulator (maximal electrical current: 15 mA), 5 participants had to be excluded from the study. Data from the remaining 34 right-handed subjects (14 male, age: 25.34 ± 3.87 years) were included into the analyses. Exclusion criteria comprised acute and history of recurrent or chronic pain, neurological or psychiatric disorders, diabetes and no normal or corrected-to-normal vision based on self-report. All female participants used hormonal contraceptives. None of the participants showed any clinically relevant scores regarding anxiety or depression as assessed with standardized questionnaires.

Participants were informed that the purpose of the study was to investigate visual perception and processing during the perception of painful electrical stimulation. Please note that subjects were only informed about a potential association of conditioned stimuli (CS) and unconditioned stimuli (US) but not of the exact contingencies between specific CS and US. They were not informed about the absence of US in the extinction phase or the implementation of unannounced US during reinstatement.

### Experimental procedures

**Fig 1:**
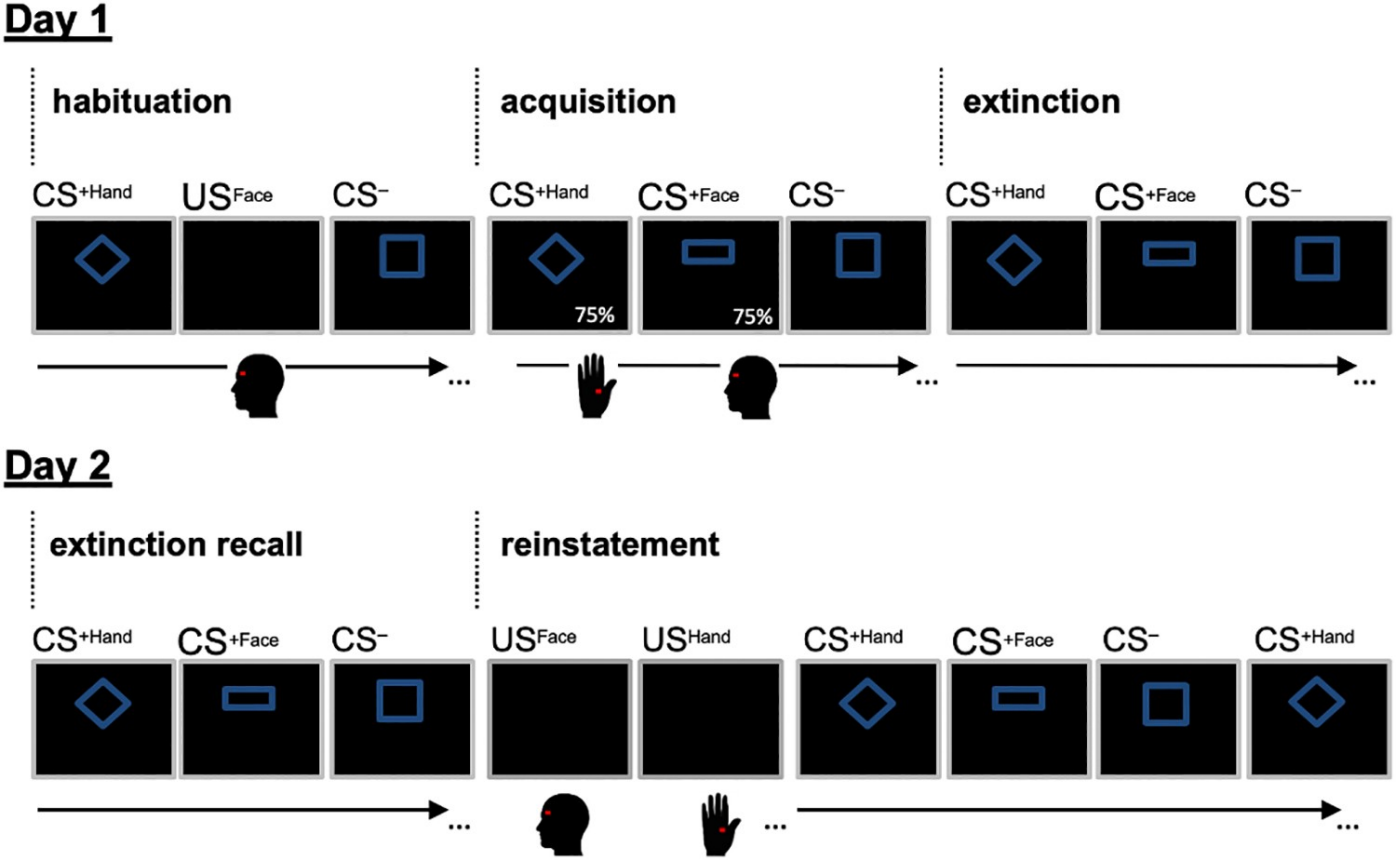
Emotional conditioning paradigm. Day 1 comprised a habituation, acquisition, and extinction phase. Day 2 comprised the extinction recall and reinstatement phases. Assignment of geometric figures to experimental conditions was randomized (example shown here). During habituation, each CS and each US was presented once. During acquisition, reinforcement rate was 75% (CS^+^ followed by US). No US were presented during extinction or extinction recall. At the beginning of the reinstatement phase, each US was presented three times without announcement. Afterwards, no US were presented.

The study was performed on two consecutive days. The conditioning paradigm (for details see below) consisted of 5 phases: 3 on day 1 (habituation, acquisition, extinction) and 2 on day 2 (extinction recall, reinstatement, see Fig 1). Changes in CS perception (i.e. valence ratings) were assessed using a 0–100 visual analog scale (VAS) with the question “How do you perceive this figure?” (anchors: “very pleasant–“neutral”–“very unpleasant”).

Please note that we chose to focus on cue valence ratings for this study to capture emotional aspects of pain-related learning, in line with previous studies from our group [35, 36] and in the literature [37].

In addition, we assessed contingency awareness separately from valence ratings after the acquisition and extinction phases, aiming to avoid an emphasis on the CS-US connection in this uninstructed learning paradigm.

Contingency awareness of the coupling between CS and US was assessed by a 0–100 VAS asking the question “How often was this figure paired with painful stimulation?” (anchors: “100% Face pain”–“never”–“100% Hand pain”). Pain intensity ratings were provided on a 0–100 VAS asking “How painful was this stimulus?” (anchors: “not painful at all”–“unbearably painful”). All VAS cursor positions had a random start position between VAS 25 and VAS 75.

On the first day, participants completed preparatory procedures, including the assessment of pain thresholds and calibration of electrical pain stimuli on both body sites. Electrical pain stimuli on the left side of the forehead (termed “face” throughout the manuscript) and the back of the left hand (termed “hand” throughout the manuscript) were adjusted separately to yield pain intensities of 70 on a 0–100 VAS.

Painful electrical stimulation was applied with two identical electrical stimulators (Digitimer DS7A constant current stimulator, Hertfordshire, UK) and surface electrodes (Specialty Developments, Bexley, UK) with a diameter of approximately 5mm that were attached to the skin using medical tape. One electrical stimulator was attached to the back of the left hand approximately 2cm under the knuckle of the index finger (dermatome C6), while the other electrode was positioned to the left side of the forehead 1cm above the outer edge of the eyebrow. For each US, we applied 15 single pulses of 0.5ms duration with an inter pulse interval of 30ms resulting in a train of painful stimulation with 0.5s duration.

Moreover, participants completed pain-related and general questionnaires (see below). After that, the first part of the conditioning paradigm (habituation, acquisition, and extinction phase) was conducted. On the second day, participants underwent the extinction recall and reinstatement phase of the conditioning paradigm.

*Day 1*. On day 1, the participants completed questionnaires assessing anxiety, depression and pain-related psychological processing. Personality states and traits have been shown to influence pain perception, chronification, and pain-related learning [38]. Participants completed the following questionnaires: (1) Pain Anxiety Symptom Scale: PASS-D [39], German version [40]; (3) Pain Catastrophizing Scale: PCS [41], German version [42]; (4) Center for Epidemiological Studies Depression Scale [43], German version: ADS-K [44]; and (5) State Trait Anxiety Inventory: STAI [45], German version [46]. All questionnaires were analyzed following their respective manuals. In order not to influence trait and state pain-related cognitions by experimentally induced pain during the experiment, questionnaires were completed before any painful stimulation was applied.

Afterwards, participants were familiarized with the electrical stimulation. First, electrical pain thresholds were assessed separately for both sites [(1) back of the left hand, (2) left side of the forehead]. Single-pulse stimuli with 0.5 ms duration were applied while increasing the current by 0.1 mA between consecutive stimuli starting at 0 mA (ascending method of limits) [47]. In order to avoid tissue damage, the upper limit was set to 15 mA. Subjects verbally indicated a change in perception from a tingling to a painful sensation (pain threshold). The assessment of pain thresholds was repeated three times for each stimulation site. The mean electrical current (mA) was defined as the site-specific pain threshold. Following, electrical pain stimuli were calibrated individually for each subject and each body site to determine the stimulation intensity corresponding to a level of 70 on a 0–100 VAS. Here, trains of electrical stimuli (duration 0.5s) of varying intensity levels around the individual pain threshold were applied. Subjects rated each stimulus regarding the pain intensity on a VAS presented on a computer screen. The stimulation intensity corresponding to a VAS level of 70 was determined for application during the experiment. All calibration procedures were assessed separately for hand and face in counterbalanced order. Subsequently, the first day of the conditioning paradigm was conducted (approximately 45 minutes task duration).

*Day 2*. On the second day of the experiment (23–25 h later), subjects performed the second part of the conditioning paradigm. We chose to perform extinction recall and the reinstatement phase on day 2 in order to allow an over-night consolidation of conditioned emotions and extinction memory [48, 49]. Note that pain intensities were not recalibrated and that electrodes were positioned at the same locations (see above/experimental procedures).

### Emotional conditioning paradigm

The emotional conditioning paradigm consisted of 5 phases: habituation, acquisition, extinction, extinction recall, and reinstatement. Three neutral visual cues (rectangle, square, and rhombus) served as conditioned stimuli. The cues were presented on a computer screen with softened edges in blue color on a black background (square: visual angle 4.99° x 4.99°, rectangle: visual angle 8.3° x 3.14°, diamond: visual angle 7.38° x 5.36°). The presentation of visual stimuli, application of electrical stimuli, and recording of the behavioral data were performed using the software Presentation (www.neurobs.com).

#### Habituation phase

Before *habituation*, subjects answered three questions on a VAS regarding general arousal (“How tense do you feel at the moment?”, anchors: “not tense at all”–“extremely tense”) and pain-related fear (“How fearful are you about the upcoming pain stimulation?”, anchors: “not fearful at all”–“extremely fearful”) separately for both body sites. Afterwards, subjects were presented three different visual cues, which later served as CS. During the habituation phase, each visual cue was presented once with a duration of 9s without any painful stimulation. Subjects rated each figure on a VAS regarding their *valence*. The VAS was shown on the computer screen during the first 7.5s of the CS presentation. Each electrical pain stimulus (US_Hand_ and US_Face_) was applied once with a duration of 0.5s and subjects rated the *pain intensity* on a VAS. Presentation of visual cues and pain stimuli was performed in a randomized order.

#### Acquisition phase

In the *acquisition* phase, the CS were pseudo-randomly assigned to one of the three experimental conditions (i.e., CS^-^, CS^+Hand^, CS^+Face^). Trials were presented in a pseudo-randomized order with no more than 3 trials of the same condition presented consecutively and the first and last CS^+Hand^ and CS^+Face^ reinforced with a US. Conditions were equally distributed within the first and second half of the acquisition phase. Each CS was presented 16 times for 9s (total: 48 trials). The CS^+Hand^ and the CS^+Face^ were partially reinforced with 0.5s painful electrical stimulation on either the hand or the face, respectively, in 75% of trials (i.e. 12 of 16 CS presentations per experimental condition). The US application started 0.5s before the end of CS presentation. The CS^-^ was never paired with a US. Inter-trial-interval (ITI) was jittered between 10 and 15s. Subjects rated their perceived *pain intensity* on every 4^th^ pain trial, resulting in three pain ratings for each body site. Pain intensity ratings were assessed in order to guarantee moderate and comparable pain intensities for face pain and hand pain stimuli throughout the acquisition phase. To ensure a constant perception of electrical pain stimuli on both body sites (VAS 70), stimulation intensities were adjusted manually by an experienced experimenter during the acquisition phase in case participants showed intense habituation or sensitization. Specifically, stimulation intensities were adjusted when VAS ratings dropped below 50 or increased to ratings above 90. Adjustments were performed in 31 participants.

Moreover, subjects rated the *valence* of the CS on every 4^th^ trial during the first 7.5s of CS presentation, resulting in four valence ratings for each CS type.

All ratings were accomplished online to ensure valid and reliable ratings [50]. Given our interest in tracking changes emotional responses to the CS (i.e. learning curves), we sampled ratings every fourth trial, providing a suitable amount of ratings for model calculation of learning curves while avoiding a possible interference with the learning process that could result from continuous or more frequent ratings.

At the end of the acquisition phase, subjects were presented one question regarding the CS-US coupling *contingency* of each CS to assess explicit learning awareness. Subjects provided answers on a 0–100 VAS. Participants were given 15 seconds to become familiar with and adjust the VAS and to confirm their contingency rating by pressing a button.

#### Extinction phase

The *extinction* phase began subsequently after these contingency ratings without any noticeable pause in between. During the extinction phase, each CS was presented 12 times without any US presentation (total: 36 trials) in order to extinguish the acquired cue-pain associations. Subjects provided CS *valence* ratings on every 4^th^ trial. ITI was 6-11s. Trials were presented in a randomized order with no more than 3 trials of the same condition presented consecutively. After the end of the extinction phase, subjects again provided *contingency* ratings regarding the CS-US coupling.

On day 2, the experiment consisted of 2 phases (approximately 15 minutes task duration).

#### Extinction recall phase

During the *extinction recall* phase, subjects were presented each CS 3 times without any US presentation (total: 9 trials) to test spontaneous recovery and extinction efficiency of pain-related emotions. Subjects were asked to rate the *valence* of 2 trials in each condition. Trials were presented in a randomized order. Importantly, US pain intensities were not recalibrated at the beginning of day 2 in order to avoid a reinstatement of pain-related emotional responses and to be able to test spontaneous recovery of pain-related emotions and extinction memory [13].

#### Reinstatement phase

Subsequently after the extinction recall phase with no noticeable pause and no further instructions, 3 unannounced US were applied to each body site in randomized order across participants. Participants rated their *pain intensity* after every US on a 0–100 VAS. Afterwards, 6 CS of each type were presented in a randomized order without any US presentation to test the *reinstatement* of previously extinguished emotional responses. Subjects provided *valence* ratings for 3 CS of each experimental condition.

### Statistical analyses

Behavioral data were automatically recorded by the software *Presentation* (https://www.neurobs.com/). The software *R* (R Studio; https://www.rstudio.com/, version 1.2.5001) was used for all behavioral analyses. One-sample t-tests were performed to compare electrical pain thresholds, calibrated intensities, pain-related fear and pain intensity ratings between both body-sites. Analyses on valence and contingency ratings were performed using linear mixed model analyses (R packages *lme4* and *lmerTest*, [51]) in order to investigate differences in learning slopes between the CS types within each experimental phase.

#### Valence ratings

Analyses were performed to test for changes over time and body-site differences in valence ratings. Separate models were calculated for each experimental phase, i.e. (I) acquisition, (II) extinction, (III) extinction recall, and (IV) reinstatement. The valence ratings provided during habituation were included into the analysis of the acquisition phase as a baseline rating prior to CS-US coupling. This was done since the first valence rating in the acquisition phase was not provided until after three CS-US pairings. Accordingly, the extinction phase model included the last rating of the acquisition phase as a baseline, while the model for the extinction recall and reinstatement phase included the last rating of the extinction phase as baseline ratings.

#### Model calculation for the acquisition and extinction phase

To account for differences in valence ratings between CS types and their changes over time, main effects of the factors *CS type* (i.e. CS^-^, CS^+Hand^, CS^+Face^) and *time* (i.e. rating number 1–5 for habituation (1) and acquisition (2–5); rating number 1–4 for last rating of acquisition (1) and extinction (2–4)) and the interaction of both factors *CS type × time* were included as fixed effects into the model. In this study we focused on the differences in emotional conditioning between CS^+Hand^ and CS^+Face^ as well as on the individual development of all CS types (CS^+Hand^, CS^+Face^, CS^-^) and did not explicitly test differential conditioning in terms of CS^+^-CS^-^ comparisons during the acquisition and extinction phase. We therefore did not subtract CS^+^ and CS^-^ ratings but rather included all three CS types separately into the models for the data of day 1 as performed previously [20, 36]. In line with previous studies [24, 52], we have further investigated differential learning (CS+–CS^-^) for the day 2, i.e., spontaneous recovery and reinstatement (see below).

#### Model calculation for the extinction recall and reinstatement phases

To account for differences in valence ratings between CS types and their changes over time, main effects of the factors *CS type* and *time* (i.e. rating number 1–2 during extinction recall; rating number 1–3 during reinstatement) and their interaction *CS type × time* were included as fixed effects into the model. This model calculation was performed to test the following questions: (i) Do previously extinguished pain-related emotional responses spontaneously recover (extinction rating 3 vs. extinction recall rating 1) and if so, is this recovery augmented for face pain compared to hand pain?; (ii) Does extinction recall of conditioned pain-related emotional responses differ between hand and face pain (extinction recall rating 1 vs. extinction recall rating 2)?; (iii) Are conditioned pain-related emotions reinstated after unexpected US presentation in terms of changes in valence (extinction recall rating 2 vs. reinstatement rating 1)?; and (iv) Do reinstated pain-related emotions subsequently extinguish differentially for face pain and hand pain?

In addition to the analysis of non-differential learning (each CS separately), we performed further analyses on differential effects ([CS^+Hand^–CS^-^] and [CS^+Face^–CS^-^] to test spontaneous recovery and reinstatement.

For all models, we tested whether the model containing a random intercept for each participant and allowing variation for the factors *CS type*, *time*, and *subjects* by adding random slopes for these factors, improved model fit. For the acquisition and extinction phase, the factor *time* was included as a continuous factor in order to account for increases and decreases of valence ratings over the course of the experiment. For the extinction recall and the reinstatement phase only, the factor time was included into the model as a categorical factor. Here, we did not expect a continuous development of the valence ratings because of the unexpected US presentation in the beginning of the reinstatement phase, which is expected to lead to a reinstatement of pain-related emotions, i.e. sudden increases of CS^+^ valence ratings.

All models were estimated according to the restricted maximum likelihood (REML) approach. The decision for best model fit was done according to the Akaike information criterion (AIC) based on maximum likelihood (ML) estimations as indicated by ANOVAs used for model comparison. Potential covariates were included into the models for each phase separately to account for their modulating influences. These comprised the differences in pain-related fear between face and hand pain, differences in pain intensity ratings between face and hand pain during acquisition, and pain-related cognitions (i.e. pain catastrophizing) since these have been shown to influence pain-related learning [38]. All models were tested for normal distribution and heteroscedasticity of the residuals.

#### Contingency ratings

Analyses were performed to test for changes in contingency ratings between phases and body-sites. Since contingency ratings were provided on a 0–100 VAS with the anchors 0 = 100% hand pain, 50 = no pain, and 100 = 100% face pain, ratings were transformed to correspond to site-specific 0–100 scales, i.e. 0 = no pain; 100 = 100% face pain and hand pain, respectively.

Further, analyses were performed to investigate differences in contingency ratings between CS types, experimental phases, and changes between phases, i.e. the acquisition and the extinction phase. The calculated final model contained main effects for the factors *phase* (i.e. acquisition and extinction) and *CS type* and their interaction *phase × CS type* as fixed effects and random intercept for the subjects and random slopes for the factors *phase*, *CS type*, and *subjects* to allow for subject-specific variation. Again, potential covariates were included into the model to account for their modulating influences. These comprised the differences in pain-related fear between face and hand pain, differences in valence and pain intensity ratings during the habituation phase, differences in pain intensity ratings during the acquisition phase, and pain-related cognitions. Again, models were calculated according to the REML approach, model fit was compared via ML estimations using the AIC and all models were tested for normal distribution and heteroscedasticity of the residuals.

## Results

### Electrical pain thresholds and calibrated intensities

Electrical pain thresholds were significantly lower for the face compared to the hand (face: 0.96 ± 0.55 mA, hand: 1.78 ± 0.95 mA, t(33) = 7.50, p < 0.001; all mean ± standard deviation). Moreover, calibrated electrical stimuli for painful stimulation during the experiment were significantly lower for the face as compared to the hand (face: 1.44 ± 1.00 mA, hand: 2.15 ± 1.46, t(33) = 4.01, p < 0.001).

### Pain-related fear and pain intensity rating

Pain-related fear ratings, which were provided once at the beginning of the habituation phase, were significantly higher for face compared to hand pain (face: 33.53 ± 24.49, hand 27.06 ± 20.60, t(30) = 3.43, p = 0.002). Importantly, pain intensity ratings did not significantly differ between calibrated face pain and hand pain in the habituation phase (face: 64.30 ± 9.53, hand 62.46 ± 11.65, t(33) = 1.06, p = 0.271) and the acquisition phase (face: 60.10 ± 11.51, hand 57.71 ± 12.85, t(33) = 1.60, p = 0.150).

### Questionnaire results

Questionnaire data were in a normal range for all subjects. For details, please see S1 Table in S1 Appendix.

### Valence ratings

**Fig 2:**
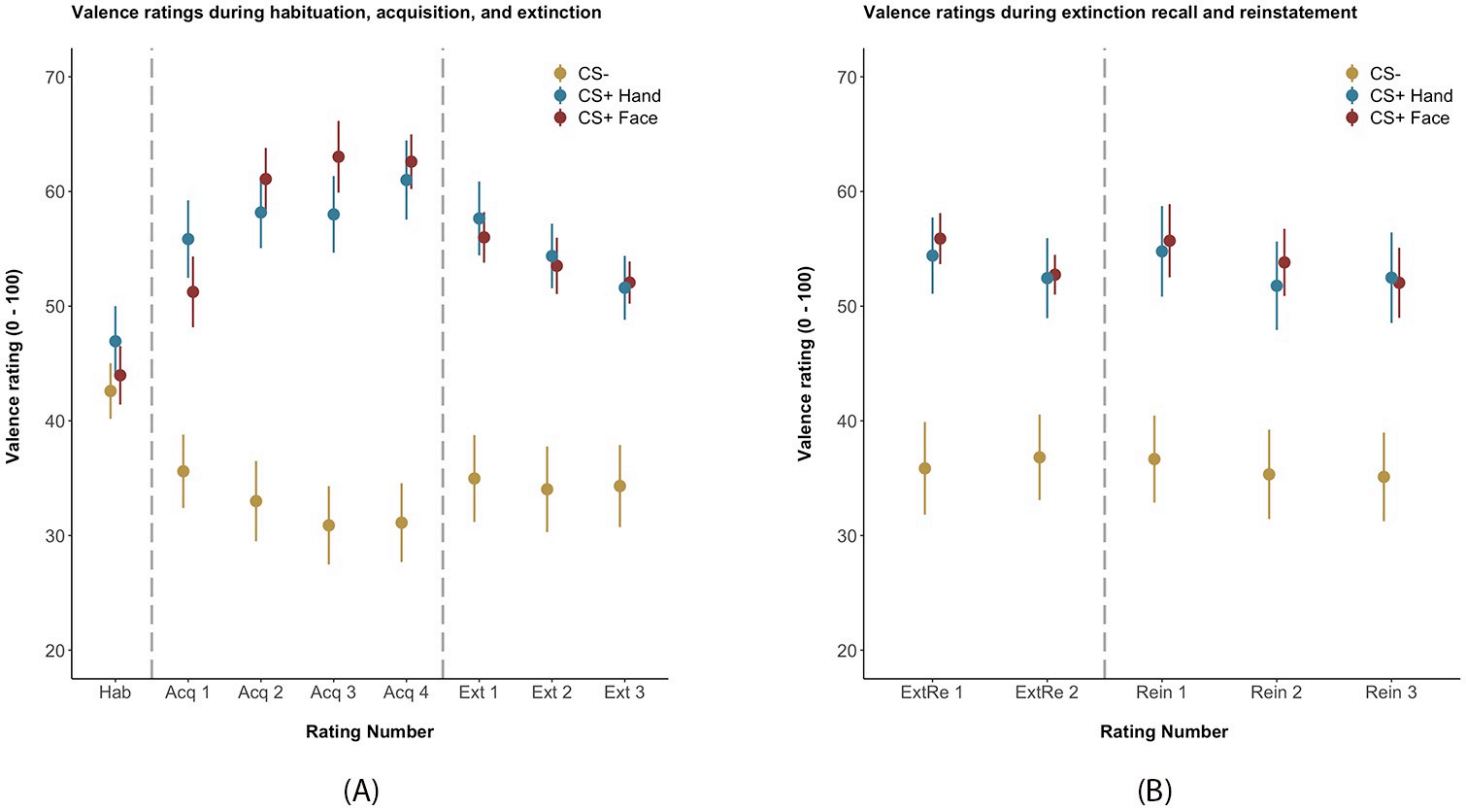
Valence ratings during the habituation (Hab), acquisition (Acq 1 –Acq 4), extinction (Ext 1 –Ext3) on day 1 of the experiment and the extinction recall (ExtRe 1 –ExtRe 2) and reinstatement phases (Rein 1 –Rein 3) on day 2 of the experiment. Ratings are given in means ± standard error of the mean. Dashed lines separate the phases. For individual data, please see S2 Fig in S1 Appendix.

Valence ratings during habituation, acquisition, and extinction are displayed in S2 Table in S1 Appendix and in Fig 2. According to the AIC, the model including random slopes for each subject and the factors *time* and *CS type* best predicted the data as compared to models without random slopes (acquisition: Δ AIC = -217.2, p < 0.001; extinction: Δ AIC = -14.9, p < 0.001; extinction recall and reinstatement: Δ AIC = -513.2, p < 0.001). This applied to all experimental phases.

The raw data in Fig 2 shows successful differential learning during the acquisition phase, i.e. increase in negative valence for both CS^+^ and an increase in positive valence for the CS^-^. Further, we observed successful extinction, i.e. decrease in negative valence for both CS^+^ and no changes in valence for the CS^-^. Please note that model calculation for valence ratings was performed for each phase separately following the same analysis pattern. Hence, results are displayed separately for each phase.

#### Acquisition phase

Results of the acquisition phase revealed a significant interaction of the factor *time* and *CS type*. Here, CS^-^ valence ratings revealed a significant main effect of the factor *time*, i.e. an increase in positive valence over the course of the acquisition phase (β: -2.92 ± 0.63, t(163.62) = -4.62, p < 0.001, d = -0.72), whereas both CS^+^ revealed a significant increase in negative valence ratings over the course of the acquisition phase (CS^+Face^: β: 4.48 ± 0.96, t(348.90) = 4.65, p < 0.001, d = 0.50; CS^+Hand^: β: 2.16 ± 0.87, t(310.67) = 2.48, p = 0.01, d = 0.28).

As an indicator for successful differential emotional learning, valence ratings for the CS^+Hand^ showed an increase in negative valence over the course of the acquisition phase as compared to valence ratings for the CS^-^ (β: 6.10 ± 0.83, t(370.82) = 7.31, p < 0.001, d = 0.76). The same result was shown for CS^+Face^ valence ratings compared to CS^-^ ratings (β: 7.80 ± 0.83, t(368.05) = 9.44, p < 0.001, d = 0.98). Confirming our hypothesis, the CS^+Face^ showed a stronger increase in negative valence over the course of the acquisition phase as compared to CS^+Hand^ (β: 1.69 ± 0.83, t(369.50) = 2.04, p = 0.04, d = 0.21). None of the potential covariates did improve model fit.

#### Extinction phase

Analysis of the extinction phase showed no significant effect of the factor *time* for the CS^-^ (β: 1.05 ± 0.72, t(98.93) = 1.45, p = 0.15, d = 0.29) indicating no significant change in valence ratings but a significant decrease of negative valence for both CS^+^ (CS^+Face^: β: -3.41 ± 0.72, t(96.80) = -4.74, p < 0.001, d = -0.96; CS^+Hand^: β: -3.12 ± 0.73, t(99.26) = -4.29, p < 0.001, d = -0.86).

Moreover, in line with our expectations, results revealed a significant interaction for the factors *time* and *CS type*. Specifically, valence ratings for the CS^+Hand^ showed a decrease over the course of the extinction phase as compared to CS^-^ valence ratings (β: -7.28 ± 0.83, t(275.72) = 5.00, p < 0.001, d = 0.60). Also, valence ratings for CS^+Face^ decreased compared to CS^-^ ratings (β: -7.87 ± 0.83, t(273.93) = 5.40, p < 0.001, d = 0.65). However, contrary to our hypothesis, valence ratings for the CS^+Face^ did not significantly differ in their decrease over the course of the extinction phase as compared to the CS^+Hand^ ratings (β: -0.29 ± 0.83, t(274.88) = -0.35, p = 0.72, d = -0.04). None of the potential covariates did improve model fit.

#### Extinction recall and reinstatement phase

Regarding a spontaneous recovery of previously extinguished emotional responses (extinction rating 4 vs. extinction recall rating 1), we observed a significant increase in negative valence for the CS^+Face^ (β: 4.26 ± 1.84, t(390.19) = 2.31, p = 0.02, d = 0.23) and an increase in negative valence at trend level for the CS^+Hand^ (β: 3.22 ± 1.84, t(390.24) = 1.75, p = 0.08, d = 0.24) indicating spontaneous recovery with comparable, small effect sizes for both CS^+^. Hence, there were no significant differences between both CS^+^ in spontaneous recovery (β: -1.04 ± 2.61, t(390.04) = -0.40, p = 0.69, d = -0.04). When investigating the differential effects between the CS^+^ and the CS^-^, we did not observe any spontaneous recovery for neither CS^+^ as indicated by non-significant differences between the ratings at the end of the extinction phase and the beginning of the extinction recall phase (CS^+Face^: β: 2.89 ± 2.24, t(177.59) = 1.29, p = 0.20, d = 0.19; CS^+Hand^: β: 0.33 ± 2.24, t(180.17) = 0.15, p = 0.88, d = 0.02). There were no significant differences between both CS^+^ (β: -2.56 ± 3.07, t(258.37) = -0.083, p = 0.41, d = -0.10). Moreover, during extinction recall, there were no significant effects of the factor *time* nor any interactions of the factor *time* and *CS type* indicating no further extinction.

We observed a significant, yet small, reinstatement effect (i.e. last extinction recall rating vs. first reinstatement rating) for the CS^+Face^ as indicated by a significant increase in negative valence after unexpected US presentation (β: 3.94 ± 1.83, t(390.17) = 2.15, p = 0.03, d = 0.22). This effect could also be observed at a trend level for the CS^+Hand^ (β: 3.46 ± 1.87, t(390.08) = 1.86, p = 0.06, d = 0.19). There were no significant differences between both CS^+^ in reinstatement and no effects for the factor *time* or interactions of the factor *time* and *CS type* within the reinstatement phase. In terms of differential effects (CS^+^–CS^-^), we observed no significant reinstatement effects (CS^+Face^: β: 2.48 ± 2.19, t(234.97) = 1.13, p = 0.26, d = 0.15; CS^+Hand^: β: -0.15 ± 2.19, t(235.52) = -0.07, p = 0.95, d = -0.01). There were no significant differences between both CS^+^ (β: -1.33 ± 3.08, t(260.0) = -0.43, p = 0.67, d = -0.05).

For day 2, we observed a significant main effect of the factor *CS type*. Ratings for both CS^+^ were significantly higher as compared to the CS^-^ (CS^+Face^: β: 16.11 ± 5.47, t(31.70) = 2.95, p = 0.006, d = 1.05; CS^+Hand^: β: 15.67 ± 6.27, t(30.18) = 2.50, p = 0.02, d = 0.91). There were no significant differences between CS^+Face^ and CS^+Hand^ valence ratings (β: -0.44 ± 3.43; t(44.74) = -0.13, p = 0.89, d = -0.04) and no interactions with the factor *time*.

None of the potential covariates did improve model fit.

### Contingency ratings

**Fig 3:**
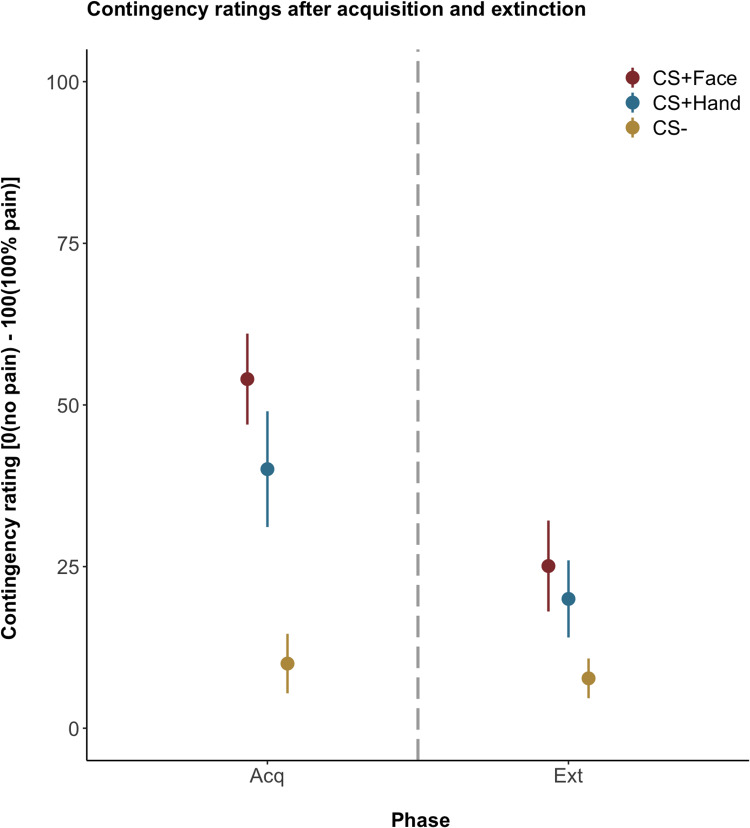
Contingency ratings after the acquisition (Acq) and extinction (Ext) phases on day 1. Ratings are given in means ± standard error of the mean. Dashed line separates the phases. For individual data, please see S3 Fig in S1 Appendix.

For contingency ratings, please see S3 Table in S1 Appendix and Fig 3. According to the AIC, the model including random slopes for the subjects and the factors *phase* and *CS type* predicted the data best as compared to models without random slopes (Δ AIC = -36.4, p < 0.001).

In general, participants correctly identified the CS to predict either hand pain, face pain, or the absence of pain. Analyses revealed a significant main effect for the factor *phase* indicating a decrease in contingency ratings from acquisition to extinction for the CS^+Face^ (β: -28.9 ± 6.00, t(109.15) = -4.82, p < 0.001, d = -0.92) and CS^+Hand^ (β: -21.42 ± 6.60, t(118.85) = -3.24, p = 0.002, d = -0.60) but not for the CS^-^ (β: -2.29 ± 6.00, t(109.15) = -0.38, p = 0.71, d = - 0.07). There was no significant interaction of the factors *phase* and *CS type* between the CS^+Face^ and the CS^+Hand^ (β: 7.50 ± 8.45, t(101.45) = 0.89, p = 0.38, d = 0.18) indicating that contingency ratings for both CS^+^ types showed a similar amount of decrease from acquisition to extinction. We observed a significant main effect for the factor *CS type*. Both, the CS^+Face^ and the CS^+Hand^, differed significantly from the CS^-^ (CS^+Face^: β: 44.00 ± 8.13, t(57.16) = 5.41, p < 0.001, d = 1.43; CS^+Hand^: β: 29.92 ± 9.32, t(54.70) = 3.21, p = 0.002, d = 0.89). Moreover, a trend for a main effect of the factor *CS type* revealed higher contingency ratings with a medium effect size for the CS^+Face^ as compared to the CS^+Hand^ (β: -14.08 ± 7.70, t(64.45) = -1.83, p = 0.07, d = -0.46).

None of the potential covariates (see *Statistical analyses*) did improve model fit. Covariates were therefore not included into the model.

## Discussion

This study investigated potential differences in the acquisition, extinction, extinction recall and reinstatement of hand and face pain-related emotional responses in healthy young volunteers in a 2-day-paradigm. Experimental electrical pain (US) applied to the hand or the face was paired with US-specific visual cues (CS) to investigate body site-specific differences in pain-related learning within the same pain modality. Confirming our hypothesis of differences in pain-related learning between hand and face pain, we found increased acquisition of pain-related emotions in terms of valence and increased contingency ratings for the CS^+^ paired with face pain compared to the CS^+^ paired with hand pain. Further, we observed small, yet not statistically significant spontaneous recovery and reinstatement effects of these pain-related emotions for face but not for hand pain.

### Successful learning during the acquisition and extinction phase

For the acquisition phase, we show successful learning of emotional responses for both CS^+^ compared to the CS^-^. In detail, valence ratings for both CS^+^ significantly increased (i.e. more negative valence) while valence ratings for the CS^-^ significantly decreased (i.e. more positive valence), consistent with its role as a safety signal [53]. Successful learning with large effect sizes for pain-related emotions using a classical conditioning paradigm has been shown previously [36, 54, 55], but never thus far in the context of face pain.

For the extinction phase, when the CS^+^ were no longer paired with pain, negative valence ratings expectedly declined significantly for both CS^+^, which is in line with previous research [35, 56]. Valence ratings at the end of the extinction phase, however, still descriptively differed from baseline ratings during habituation, indicating that our sample of healthy participants did not show complete extinction of emotional responses in terms of valence ratings, at least not within 12 extinction trials. This resistance to extinction might be due to the biological warning function and genuinely aversive characteristic of pain as has also been observed before for interoceptive painful stimuli serving as US [31, 33, 34]. Another explanation could be that emotional responses, such as valence, do not get as easily extinguished as for instance conditioned fear ratings or predictive US expectation ratings [13, 57]. A spider phobic, for example, might not show fear after successful exposure therapy, but might still perceive a spider as unpleasant. Our finding of incomplete extinction, however, might also be a result of the repeated evaluation of CS valence during the extinction procedure, as found and discussed in a recent emotional conditioning study [58]. Further, a meta-analysis of emotional conditioning studies concluded that emotional conditioning is sensitive to extinction, although extinction of emotional responses might occur at a slower rate than what is often reported for other forms of pavlovian conditioning [59]. This is in line with our findings of significant, yet incomplete extinction. In this study, we have explicitly decided to assess valence ratings in order to focus on the emotional and affective aspects of pain [31, 32] as these might also shape pain perception [60], especially in chronic pain patients. Future studies could investigate body-site specific differences and in particular face pain-specific mechanisms of extinction learning using fear ratings or US expectation ratings [50] to shed more light on different types of learning (e.g. evaluative vs. predictive).

Our result of successful emotional conditioning in terms of acquisition and extinction are further supported by the subjects’ contingency ratings, which were higher for both CS^+^ as compared to the CS^-^, indicating that subjects were aware of the coupling of both CS^+^ with pain during the acquisition phase. Post-extinction, contingency ratings were significantly lower for both CS^+^ as compared to post-acquisition, supporting our reported findings in valence rating changes.

### Stronger acquisition but comparable extinction of pain-related negative valence for face compared to hand pain

While other studies examined fear or emotional learning using single pre- or post-acquisition or extinction ratings [23, 36, 61, 62], we were able to acquire more precise information regarding the temporal dynamics and slopes of these processes by repeatedly assessing subjective valence ratings over the course of the experiment. In line with our hypothesis, valence ratings showed a stronger negative increase over the course of the acquisition phase for the CS^+Face^ compared to the CS^+Hand^. This result indicates that face pain-related negative emotions seem to be acquired steeper and faster as compared to hand pain-related emotions, which is in line with previous research showing that face pain and pain applied to extremities positioned near the head or face are perceived as more threatening and result in increased fear compared to painful stimulation of other body parts [27–29, 63]. Although we carefully matched the intensity of both pain stimuli, the more salient face pain might have been more aversive or arousing [64] to subjects, leading to increased conditioned responses [34].

Contrary to our hypotheses, there was no difference in the slope of extinction of pain-related negative emotions between the CS^+Hand^ and the CS^+Face^. Both CS^+^ showed a comparable decrease in valence ratings. The difference in biological relevance of the two investigated body sites thus seems to affect the acquisition rather than the extinction in pain-related emotional conditioning. One might speculate that the observed body-site specific differences are only evident under certain conditions, for instance in the presence of multiple, more or less threatening stimuli, i.e. face pain and hand pain, presumably leading to a state of enhanced top-down processes such as arousal [65]. Further, top-down processes, such as a fear-related enhanced attention towards the more threatening CS might result in the observed body-site learning differences during acquisition [66, 67]. Once these stimuli are not present anymore (and with a reduced arousal or similar attention levels), the formation of new inhibitory associations (i.e. extinction learning) might work equally well for CS associated with US of different threat levels [65], at least in healthy subjects. In this study, fear of pain assessed prior to the experiment was increased for face compared to hand pain, which might have increased attention for the CS predicting face pain in relation to hand pain. Visual attention to the site of painful stimulation has been shown before [68]. Future studies might test this assumption experimentally in enhancing arousal or attention during the extinction phase, for instance by intermittently applying a (new) US in a non-contingent manner [69] and controlling individual arousal levels to test the assumption of differential arousal to multiple, more or less threatening stimuli.

### Further support for increased learning mechanisms of face pain-related emotions—Contingency ratings

As expected, contingency ratings, which directly assess perceived awareness of the predictive value of CS, were significantly higher for both CS^+^ after the acquisition phase as compared to the CS^-^, indicating largely accurate contingency awareness.

Supporting our hypothesis of increased learning for face compared to hand pain, contingency ratings were descriptively higher for the coupling of the CS^+Face^ and face pain as compared to the CS^+Hand^ and hand pain after both phases. Although reinforcement rates did not differ, subjects were more aware of the CS-US association for face compared to hand pain. Therefore, subjects not only perceived the CS^+Face^ to be more unpleasant than the CS^+Hand^ but also to predict painful stimulation more often. This result indicates that the biological relevance of face pain not only affects emotional aspects, such as valence, but also cognitive aspects, such as perceived contingency and supports the notion that contingency awareness and conditioned responses are highly related [50, 70]. From a functional perspective, successful learning about signals predicting pain, either explicitly or implicitly, may foster an adaptive way of coping with upcoming threatening stimuli [3].

Interestingly, contingency, but not valence ratings differed after the extinction phase between both pain conditions, indicating that these emotional and cognitive aspects related to pain learning may not develop uniformly and represent related, yet separate processes [31, 71]. Taken together, face pain seems to elicit higher pain-related fear [27, 63] and negative emotions, defensive responses [29], and, as shown here at least at trend level with medium effect sizes, increased contingency awareness.

In terms of cognitive awareness towards pain, a limitation of this study might be that participants filled out pain-related questionnaires prior to the conditioning experiment. This procedure was chosen to avoid influencing trait and state pain-related cognitions by receiving painful stimulation during the experiment. Answering questionnaires on pain-related thoughts and behavior might, however, have influenced the subjects’ thoughts during the conditioning task and might have primed awareness to pain in general. However, as there were no questionnaires addressing hand or face pain specifically, differential (priming) effects on hand and face pain are unlikely.

### Spontaneous recovery and reinstatement effects for pain-related emotions

The observed increase in negative valence for both CS^+^ at the beginning of the extinction recall phase suggests spontaneous recovery [12] of pain-related emotions, albeit with small effect sizes. However, when analyzing differential effects (CS+–CS^-^), we did not observe significant spontaneous recovery, but our results at least suggest a small effect on the descriptive level for face pain only. This finding might be explained by the absence of full extinction on day 1. Further, increasing the sample size might have increased the power and potentially strengthened our results. As is, these results have to be interpreted with caution as we do not observe consistent results when comparing analyses of differential and non-differential (spontaneous recovery) effects.

Moreover, according to our hypothesis, we observed small reinstatement effects of pain-related negative valence after unexpected painful stimulation of the hand and face. Neural activation during the reinstatement of pain-related fear and emotions has been assessed before using visceral US [20, 23, 36], but has never been reported at the behavioral level using two US. Again, when analyzing differential effects, we did not find significant reinstatement. Regarding effect sizes, however, our data shows a small reinstatement effect for face and no effect for hand pain. These results could once more point towards increased conditioned responses and relevance of face compared to hand pain. Increasing sample sizes or using other outcome measures (e.g. US expectancy ratings, skin conductance, startle response) may clarify this question.

### Implications for chronic pain populations

Differential fear learning has been reported to be altered in chronic pain populations [17, 18, 52]. Klinger, Matter [25], for instance, reported increased conditioned responses in tension-type headache patients as compared to healthy controls. In this study, patients suffering from headache and those suffering from chronic back pain did not show differences in the acquisition for painful US applied to the extremities. However, neither extinction nor differences in acquisition for pain applied to the head or back were investigated. In another study, patients suffering from trigeminal neuralgia showed increased defensive responses and their defensive “peripersonal space” was enhanced and enlarged ipsilateral to the affected body site [72]. Regarding reinstatement of pain-related fear and emotions, results are scarce. In chronic pain patients and healthy controls, Icenhour, Langhorst [20] report first evidence suggesting altered reinstatement processes in patients with chronic visceral pain.

It could be hypothesized that patients suffering from chronic head and face pain would show maladaptive learning, i.e., enhanced acquisition, impaired extinction and enhanced reinstatement for face pain, as compared to healthy subjects or other chronic pain populations, such as chronic back pain patients. This may contribute to the chronification of head and face pain disorders and should be investigated in future research.

## Conclusion

This study revealed a significantly increased acquisition of pain-related emotional learning for electrical pain applied to the face as compared to the hand in young healthy participants, which is in line with previous findings on increased face pain-related fear [27, 63]. In young healthy participants, there were no significant differences in extinction-related phenomena of previously acquired pain-related emotional responses for face and hand pain. However, chronic pain populations, especially patients suffering from chronic head and face pain diseases, might show different extinction mechanisms for pain-related emotions, particularly in the head and face area, which might contribute to the chronification of these pain diseases.

## Supporting information

S1 Appendix Supplementary information on the manuscript. (DOCX)

S1 Data Dataframe of the raw data day 1. (CSV)

S2 Data Dataframe of the raw data day 2. (CSV)
