# Supplementary material for: Enhanced pain-related conditioning for face compared to hand pain
Source: PLoS One. 2020 Jun 19;15(6):e0234160. doi: 10.1371/journal.pone.0234160 (PMC7304572; doi:10.1371/journal.pone.0234160)
Supplement: S1 Appendix — (DOCX) [file pone.0234160.s001.docx]

| **Questionnaire** | **Mean** | **SD** | **Range** |
| --- | --- | --- | --- |
| **PASS 1** | 1.55 | 0.64 | 0.3 – 3.0 |
| **PASS 2** | 1.93 | 0.55 | 1.0 – 3.1 |
| **PASS 3** | 0.88 | 0.48 | 0.1 – 2.4 |
| **PASS 4** | 1.10 | 0.59 | 0 – 2.2 |
| **PCS** | 13.43 | 6.45 | 4 – 25 |
| **ADS** | 9.46 | 5.27 | 0 – 20 |
| **STAI State** | 31.91 | 4.32 | 23 – 48 |
| **STAI Trait** | 33.51 | 5.76 | 23 – 49 |

S1 Table 1. Questionnaire data (Mean, standard deviation (SD) and range). PASS: Pain Anxiety Symptom Scale (4 subscales); PCS; Pain Catastrophizing Scale; ADS-K: Center for Epidemiological Studies–Depression Scale; STAI: State Trait Anxiety Inventory.

| Valence Ratings / CS Type | Hab | Acq 1 | Acq 2 | Acq 3 | Acq 4 | Ext 1 | Ext 2 | Ext 3 | ExtRe 1 | ExtRe 2 | Rein 1 | Rein 2 | Rein 3 |
| --- | --- | --- | --- | --- | --- | --- | --- | --- | --- | --- | --- | --- | --- |
| CS^-^ | 42.60 ± 14.35 | 35.60 ± 14.55 | 33.00 ± 19.80 | 30.89 ± 20.20 | 31.12 ± 20.02 | 34.97 ± 22.07 | 34.03 ± 21.78 | 34.31 ± 21.18 | 35.41 ± 20.68 | 37.26 ± 19.67 | 36.67 ± 19.71 | 34.85 ± 19.88 | 35.59 ± 20.46 |
| CS^+Hand^ | 46.94 ± 17.62 | 55.85 ± 19.45 | 58.17 ± 18.44 | 58.00 ± 19.78 | 61.00 ± 20.13 | 57.65 ± 18.82 | 54.37 ± 16.67 | 51.60 ± 16.50 | 54.25 ± 15.16 | 52.41 ± 19.84 | 54.38 ± 20.84 | 52.17 ± 19.47 | 52.58 ± 20.94 |
| CS^+Face^ | 43.97 ± 15.12 | 51.24 ± 17.76 | 61.82 ± 15.82 | 63.03 ± 18.52 | 62.60 ± 14.12 | 56.00 ± 13.05 | 53.51 ± 14.55 | 52.06 ± 10.92 | 55.12 ± 11.07 | 52.58 ± 9.89 | 55.64 ± 16.29 | 54.88 ± 15.03 | 51.14 ± 16.14 |

S1 Table 2. Mean ± SD of the raw data of valence ratings (VAS) for each experimental condition (CS Type) and timepoint. Hab = Habituation; Acq = Acquisition; Ext = Extinction; ExtRe = Extinction recall; Rein = Reinstatement.

| Contingency Ratings / CS Type | Acquisition | Extinction |
| --- | --- | --- |
| CS^-^ | 10 ± 27.22 | 7.71 ± 18.19 |
| CS^+Hand^ | 40.07 ± 48.22 | 20.00 ± 33.76 |
| CS^+Face^ | 54.00 ± 41.62 | 25.09 ± 41.61 |

S1 Table 3. Mean ± SD of the transformed raw data of contingency ratings (VAS) for each experimental condition (CS Type).


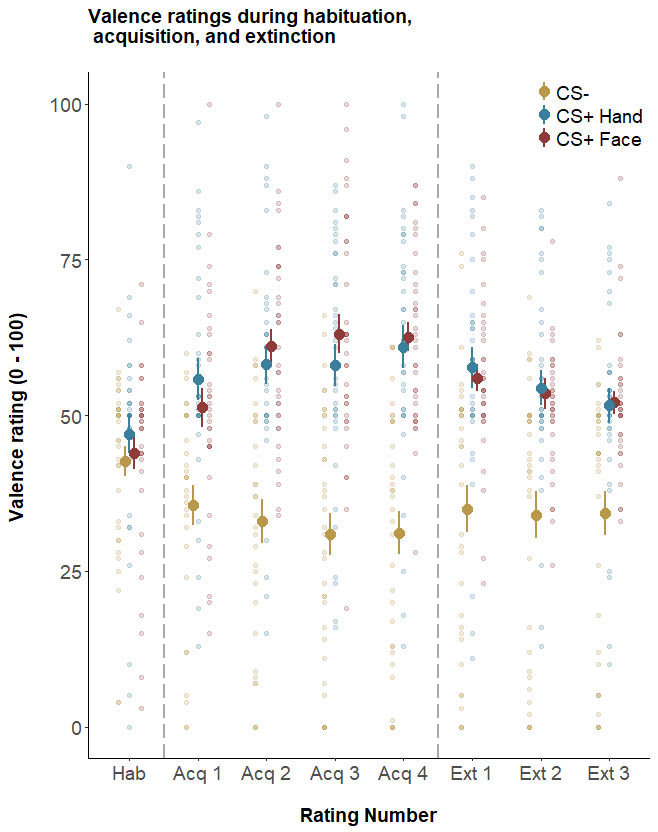


S1 Fig 1. Valence ratings during the habituation (Hab), acquisition (Acq 1 – Acq 4), and extinction phases (Ext 1 – Ext3) on day 1 of the experiment. Ratings are given in means ± standard error of the mean. Light dots indicate individual data points. Dashed lines separate the phases.


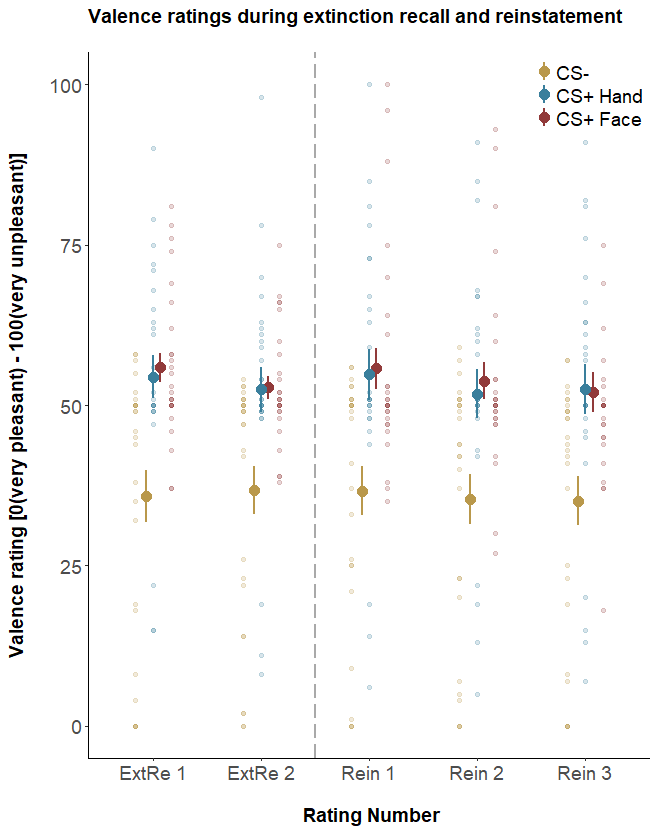


S1 Fig 2. Valence ratings during the extinction recall (ExtRe 1 – ExtRe 2) and reinstatement phases (Rein 1 – Rein 3) on day 2 of the experiment. Ratings are given in means ± standard error of the mean. Light dots indicate individual data points. Dashed line separates the phases.


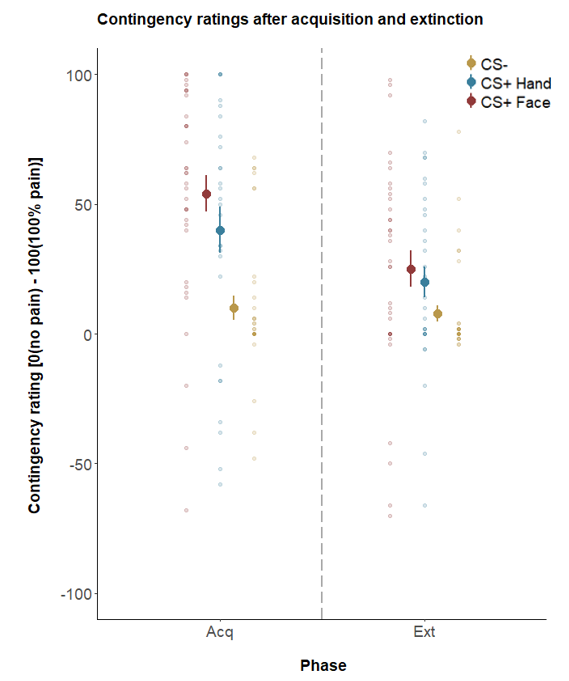


S1 Fig 3. Contingency ratings after the acquisition and extinction phases on day 1. Ratings are given in means ± standard error of the mean. Please note that following the transformation procedure as described in “Contingency ratings”, for both CS^+^, negative values indicate a falsely perceived association of CS and US (e.g. CS^+Hand^ paired with face pain). For the CS^-^, positive values indicate a perceived association with hand pain, whereas negative values indicate a perceived association with face pain. Light dots indicate individual data points. Dashed line separates the phases.

**Influence of contingency ratings on valence ratings**

We performed additional models on valence ratings that included the factor *contingency* to investigate the influence of contingency awareness on the acquisition and extinction of pain-related emotions, i.e. valence ratings of CS types. These models only differed from those described in the manuscript in including the factor *contingency*.

For the *acquisition* phase, including the contingency ratings as a covariate into the analyses of valence ratings revealed a significant interaction of the factors *time* and *contingency* for the CS^+Hand^ only, indicating stronger increases in negative valence ratings with increased contingency awareness (estimated parameters: 0.03 ± 0.01; t(330.25) = 2.47, p = 0.01, d = 0.27). This interaction could not be observed for the CS^-^ (estimated parameters: 0.01 ± 0.02; t(364.92) = 0.71, p = 0.43, d = 0.09) or the CS^+Face^ (estimated parameters: 0.004 ± 0.01; t(335.63) = 0.3, p = 0.76, d = 0.03). For the *extinction* phase, there was no interaction between the changes in valence ratings over time and the contingency awareness for any of the CS types (CS^+Hand^: estimated parameters: -0.04 ± 0.02; t(253.50) = -1.74, p = 0.08, d = -0.22; CS^+Face^: estimated parameters: -0.02 ± 0.02; t(245.96) = -1.48, p = 0.14, d = -0.19; CS^-^: estimated parameters: -0.05 ± 0.04; t(272.33) = -1.38, p = 0.17, d = -0.17).

It would be tempting to speculate that the higher fear of face pain (1, 2) and face pain-related emotions can induce pronounced evaluative learning for face pain that might not necessarily depend on conscious awareness. A similar phenomenon has been shown in a previous neuroimaging study, where we found enhanced neural (re)activation during a memory encoding and recognition task for visual stimuli that were paired with face as compared to hand pain although behavioural memory performance did not differ between both experimental conditions (1, 3).

**References**

1. Schmidt K, Forkmann K, Sinke C, Gratz M, Bitz A, Bingel U. The differential effect of trigeminal vs. peripheral pain stimulation on visual processing and memory encoding is influenced by pain-related fear. NeuroImage. 2016.

2. Schmidt K, Schunke O, Forkmann K, Bingel U. Enhanced Short-Term Sensitization of Facial Compared With Limb Heat Pain. The journal of pain : official journal of the American Pain Society. 2015;16(8):781-90.

3. Schmidt K, Forkmann K, Schultz H, Gratz M, Bitz A, Wiech K, et al. Enhanced neural reinstatement for evoked facial pain compared to evoked hand pain. The journal of pain : official journal of the American Pain Society. 2019.
